# Supplementary material for: Live Fluorescence Imaging of F-Actin Organization in Chick Whole Embryo Cultures Using SiR-Actin
Source: Cells. 2021 Jun 22;10(7):1578. doi: 10.3390/cells10071578 (PMC8303455; doi:10.3390/cells10071578)
Supplement: Supplementary file 1 [file cells-10-01578-s001.zip › cells-1189677 supplementary.pdf]

SUPPLEMENTARY MATERIAL

Movie S1_Young embryo 240nM SiR-actin

Darkfield and widefield fluorescent time lapse movie of chicken embryo cultured *in vitro* in a submerged filter paper sandwich in the presence of 240nM SiR-actin in the culture medium. Development progressed from the 3-somite stage embryo (HH7 up to 10-somite stage (HH10) during 12 hours of incubation.

Movie S1 Young embryo 240nM SiR-actin

Video URL: **https://youtu.be/DtfjRQWlqvg**

Movie S2_240 nM SiR-actin high magnification

Formation of somites in chicken embryo, pre-incubated with 240nM SiR-actin overnight prior to imaging.

Movie S2 240 nM SiR-actin high magnification

Video URL: **https://youtu.be/61e4w6mynZI**

Movie S3_older embryo 50nM SiR-actin

Darkfield and widefield fluorescent time lapse movie of chicken embryo cultured *in vitro* in a submerged filter paper sandwich in the presence of 50nM SiR-actin in the culture medium. Developed progressed from the 8-somite stage embryo (HH9 to 9+) up to 21-somite stage (HH13-14) during 28 hours of incubation.

Movie S3 older embryo 50nM SiR-actin

Video URL: **https://youtu.be/ YcHdMUbtQg**

Movie S4_SiR-actin stretched embryo

DF and WF microscopic time lapse images of the segmenting paraxial mesoderm in a stretched chicken embryo cultured *in vitro* in a submerged filter paper sandwich in presence of 50 nM SiR-actin. The movie starts after the end of stretching protocol.

Movie S4 SiR-actin stretched embryo

Video URL: **https://youtu.be/2b-gCWH-Gf0**

Table S1. Microscope settings Leica SP5. Overview of settings used to acquire micrograph presented in Figure 3 B (left table) and Figure 3 C (right table).

Dimension	Logical Size	Physical Length	Physical Design	Dimension	Logical Size	Physical Length	Physical Design
X	1024	492.05 µm	0 µm	X	1024	580.48 µm	0 µm
Y	1024	492.05 µm	0 µm	Y	1024	580.48 µm	0 µm
T	5	8m0.000 s	0 s	T	24	40m0.000 s	0 s

**Scanner Settings**		**Scanner Settings**	
UV Shutter	0	UV Shutter	0
Visible Shutter	1	Visible Shutter	1
Scan Mode	xyt	Scan Mode	xyt
Pinhole (m)	50 µm	Pinhole (m)	110 µm
Pinhole (airy)	896.83	Pinhole (airy)	1.97
Size-Width	492.06 µm	Size-Width	590.48 µm
Size-Height	492.06 µm	Size-Height	590.48 µm
Voxel-Width	481 nm	Voxel-Width	577.2 nm
Voxel-Height	481 nm	Voxel-Height	577.2 nm
Zoom	3	Zoom	2.5
Scan-Direction	2	Scan-Direction	2
Sequential Mode	0	Sequential Mode	0
Frame Accumulation	1	Frame Accumulation	1
Frame Average	1	Frame Average	1
Line Average	3	Line Average	5
Resolution	8 bits	Resolution	8 bits
Channels	2	Channels	2
Format-Width	1024 pixels	Format-Width	1024 pixels
Format-Height	1024 pixels	Format-Height	1024 pixels
Line-Accumulation	1	Line-Accumulation	1
Iterations	1000	Iterations	1000
Size-Depth	480 s	Size-Depth	2.8 ks
AOTF(405)	0.00 %	AOTF(405)	0.00 %
AOTF(458)	0.00 %	AOTF(458)	0.00 %
AOTF(476)	0.00 %	AOTF(476)	0.00 %
AOTF(488)	0.00 %	AOTF(488)	0.00 %
AOTF(496)	0.00 %	AOTF(496)	0.00 %
AOTF(514)	0.00 %	AOTF(514)	0.00 %
AOTF(561)	0.00 %	AOTF(561)	0.00 %
AOTF(633)	20.00%	AOTF(633)	13.00%
HyD 1	Active	HyD 1	Active
HyD 1 (Gain)	61.0	HyD 1 (Gain)	61.0
HyD 1 (AcquisitionMode)	Standard	HyD 1 (AcquisitionMode)	Standard
HyD 1 (OverloadState)	OK	HyD 1 (OverloadState)	OK
PMT 2	Active	PMT 2	Active
PMT 2 (Offs)	0.0 %	PMT 2 (Offs)	0.0 %
PMT 2 (HV)	0.0	PMT 2 (HV)	0.0
PMT 2 (HV_Unit)	V	PMT 2 (HV_Unit)	V
PMT 2 (Preamp)	Direct	PMT 2 (Preamp)	Direct
PMT 3	Inactive	PMT 3	Inactive
System Number	5100001274	System Number	5100001274
Laser (405 Diode, UV)	Off	Laser (405 Diode, UV)	Off
Laser (Argon, visible)	Off	Laser (Argon, visible)	Off
Laser (Argon, visible) (Power)	-11 %	Laser (Argon, visible) (Power)	-11 %
Laser (DPSS 561, visible)	On	Laser (DPSS 561, visible)	On

Laser (HeNe 633, visible)	On	Laser (HeNe 633, visible)	On
Scan Field Rotation	8 degrees	Scan Field Rotation	8 degrees
Scan Speed	200 Hz	Scan Speed	200 Hz
Objective	10.0x0.40 DRY UV	Objective	10.0x0.40 DRY UV
Numerical aperture (Obj.)	400	Numerical aperture (Obj.)	400
Refraction index	1	Refraction index	1
DM6000 Stage Pos x	0.01279385575423	DM6000 Stage Pos x	0.01284464186101
DM6000 Stage Pos 7	0.01040080522166	DM6000 Stage Pos 7	0.01033960896393
Emission bandwidth PMT 2: begin - end	782nm – 790nm	Emission bandwidth PMT 2: begin - end	782nm – 790nm

Table S2. Microscope settings Leica SP5. Overview of settings used to acquire micrograph presented in Figure 3 D.

**Dimension**	**Logical Size**	**Physical Length**	**Physical Design**
X	512	234.32 µm	0 µm
Y	512	234.32 µm	0 µm

**Scanner Settings**	
UV Shutter	0
Visible Shutter	1
Scan Mode	xyt
Pinhole (m)	600 µm
Pinhole (airy)	3.85
Size-Width	234.32 µm
Size-Height	234.32 µm
Size-Depth	0 µm
StepSize	0.05 µm
Voxel-Width	458.5 nm
Voxel-Height	458.5 nm
Zoom	1
Scan-Direction	2
Sequential Mode	0
Frame Accumulation	1
Frame Average	1
Line Average	1
Resolution	8 bits
Channels	2
Format-Width	512 pixels
Format-Height	512 pixels
Line-Accumulation	1
Section	1
AOTF(405)	0.00 %
AOTF(458)	0.00 %
AOTF(476)	0.00 %
AOTF(488)	0.00 %
AOTF(496)	0.00 %
AOTF(514)	0.00 %
AOTF(561)	0.00 %
AOTF(633)	25.00%
HyD 1	Active
HyD 1 (Gain)	50.0

HyD 1 (AcquisitionMode)	Standard
HyD 1 (OverloadState)	OK
PMT 2	Active
PMT 2 (Offs)	0.0 %
PMT 2 (HV)	0.0
PMT 2 (HV_Unit)	V
PMT 2 (Preamp)	Direct
PMT 3	Inactive
System Number	5100001274
Laser (405 Diode, UV)	Off
Laser (Argon, visible)	Off
Laser (Argon, visible) (Power)	-11 %
Laser (DPSS 561, visible)	On
Laser (HeNe 633, visible)	On
Scan Field Rotation	0 degrees
Scan Speed	100 Hz
Objective	HCX APO L U-V-I 63.0x0.90 Water UV
Numerical aperture (Obj.)	900
Refraction index	1.33
DM6000 Stage Pos x	0.01261429041648
DM6000 Stage Pos 7	0.01029187949618
Emission bandwidth PMT 2: begin - end	782nm – 790nm

Table S3. Average somite formation time. Number of somites that were completely separated from the anterior tip of the PSM were counted at selected time-points during imaging. Darkfield microscopic time lapse images were used.

	Sample	SiR-actin concentration [nM]	No. somites formed	Elapsed time [min]	Average time/somite [min]
**Control**	**1**	**0**	**11**	**770**	**77**
	**2**	**0**	**16**	**1300**	**86.7**
	**3**	**0**	**19**	**1329**	**73.8**
**SiR**	**1**	**50**	**14**	**1166**	**89.7**
	**2**	**50**	**15**	**1130**	**80.7**
	**3**	**240**	**9**	**711**	**88.9**

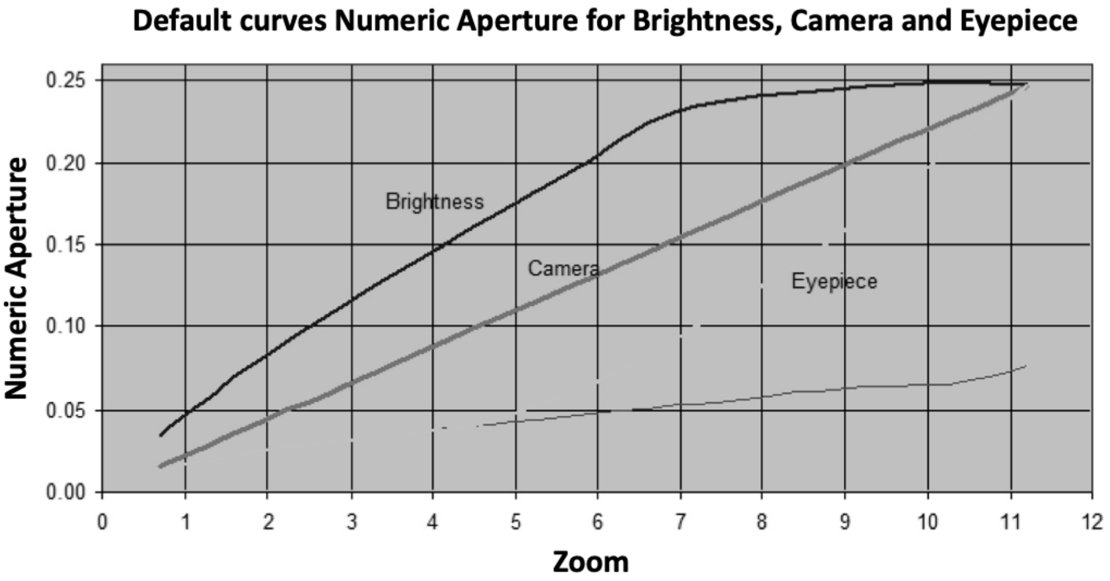

Figure S1. Numerical aperture Zeiss PlanNeofluar 1.0x objective. NA of objective in dependence of zoom value as received as information from Zeiss (black line).

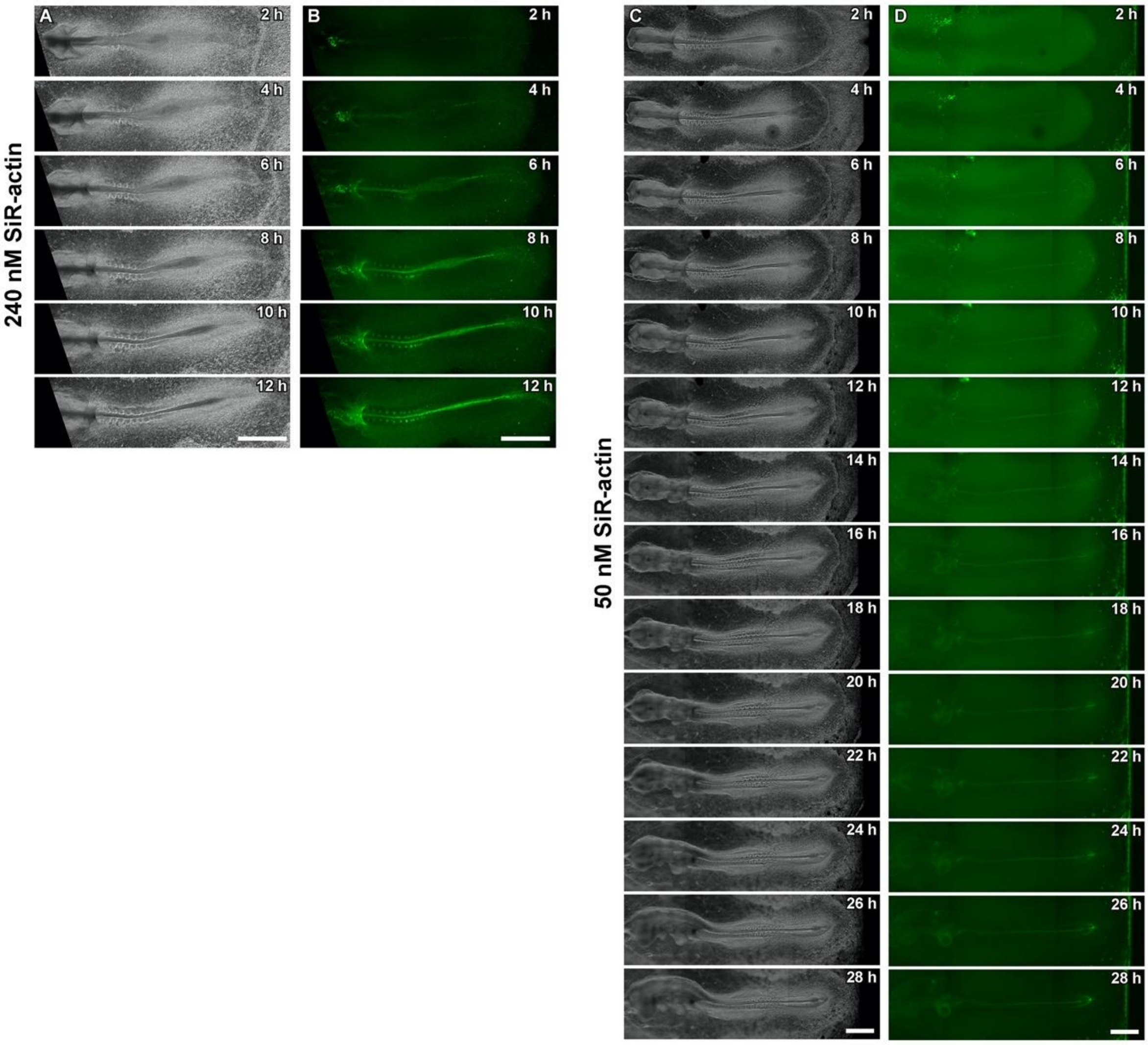

Figure S2. Embryonic development and gradual increase of SiR-actin fluorescence in chick embryos cultured ex ovo with 240 nM SiR-actin (left) and 50 nM SiR-actin (right) at intervals of two hours. .
